# Steroids in Central Retinal Vein Occlusion: Is There a Role in Current Treatment Practice?

**DOI:** 10.1155/2015/594615

**Published:** 2015-10-08

**Authors:** Mohammed Ashraf, Ahmed A. R. Souka

**Affiliations:** Ophthalmology Department, Faculty of Medicine, Khartoum Square, El Azarita, Alexandria, Egypt

## Abstract

With the current widespread use of anti-VEGFs in the treatment of central retinal vein occlusion (CRVO), the role for steroids has become greatly diminished. Recent large scale randomized control trials (RCTs) have established the efficacy and safety of anti-VEGFs in the treatment of CRVO. Steroids are known to cause elevations in intraocular pressure as well as increase the risk of cataract formation. With that in mind many ophthalmologists are injecting steroids less frequently. This paper aims to review some of the data pertaining to the use of steroids either as a first line monotherapy, adjunct therapy, or an alternative therapy to help answer the question: Is there currently any role for steroids in the management of CRVO?

## 1. Introduction

Macular edema is a frequent cause of decreased visual acuity in patients with central retinal vein occlusion [1, 2]. The mechanism of macular edema (ME) in central retinal vein occlusion (CRVO) is not completely understood. One of the main factors in the development of macular edema apart from increased venous pressure is the elevated level of vascular endothelial factor (VEGF) with subsequent break down of the blood-retinal barrier and leakage [3–5]. A minor role exists for upregulation of other inflammatory mediators (interleukin- (IL-) 1*β*, IL-2, IL-5, IL-8, IL-9, IL-10, IL-12, IL-13, eotaxin, granulocyte colony stimulating factor, interferon-inducible 10 kDa protein, monocyte chemotactic protein-1, and interferon-*γ*) as well as dysregulation of endothelial tight junctions [6–8]. The current standard of treatment based on several large randomized control trials (RCTs) is anti-VEGF injections [9, 10]. Intravitreal anti-VEGF injections target VEGF in the vitreous cavity. Corticosteroids work by a different mechanism, mainly targeting the inflammatory pathway. Corticosteroids decrease expression of VEGF and several other inflammatory mediators and therefore may indirectly reduce VEGF levels in the vitreous cavity [6, 11, 12]. Corticosteroids also have an anti-inflammatory role reducing vascular permeability, inhibiting leukocyte movement, suppressing homing and migration of inflammatory cells, stabilizing endothelial tight junctions, and inhibiting prostaglandins and other cytokines [13–15].

It is important to note that whilst anti-VEGFs like ranibizumab and bevacizumab target VEGF already present in the vitreous, corticosteroids affect the expression and production of VEGF as well as other mediators. These differences may explain some of the differences in outcomes between the two drugs. In addition it would seem that their mechanisms of action would seem synergistic and not competitive to one another.

## 2. Steroids as Monotherapy

There are two major steroids that are currently being used in the treatment of central retinal vein occlusion: triamcinolone acetonide and the dexamethasone implant. Both these drugs were studied in two large randomized controlled trials SCORE [16] for triamcinolone and GENEVA [17] for the dexamethasone implant.

### 2.1. SCORE Study

This was one of the first large scale RCTs that looked into the effectiveness of steroids as a treatment regimen. Before the use of steroids the standard of care was observation, particularly after grid laser photocoagulation proved its ineffectiveness in the CVOS study [18]. The SCORE study was a multicenter randomized clinical trial that included 271 participants randomized into 3 groups: observation, 1 mg triamcinolone, and 4 mg triamcinolone groups [16]. The study used a preservative-free, nondispersive formulation to avoid a postinjection inflammatory reaction (Allergan Inc., Irvine, California; 4 mg brand name, Trivaris). This drug is not available in common clinical practice and the more commonly used Kenalog (Bristol-Myers, Squibb, Princeton, New Jersey, Alcon Inc., Fort Worth, Texas) was not evaluated in this large RCT. Patients were injected on their initial visit and retreated at 4-month intervals according to their originally assigned treatments.

Patients in the observation group were not purely naïve in that they could receive intravitreal triamcinolone when there was loss of 15 letters or more present on 2 consecutive 4-month intervals. The data from these patients was analyzed in the observation group which means that it might have altered some of the final results and indirectly the comparison between the two groups.

#### 2.1.1. Baseline Criteria (Table 1)

To help put the results of this study in their proper context it is important to understand the major differences between SCORE and another major study, the CRUISE study [9]. A summary of the major differences in baseline criteria is shown in Table 1. The CRUISE study helped establish Ranibizumab and anti-VEGFs as the preferred treatment modality for CRVO. With regard to baseline criteria the average duration of edema was 4 months, with 29% of participants having edema for less than 3 months, 81% of patients having edema for 6 months or less, and 19% of patients having an edema for more than 7 months. For CRUISE, the mean duration was 3.3 months with duration of <3 months in 69% of patients [9]. The mean baseline VA was 51 letters (Snellen equivalent, approximately 20/100) in SCORE, compared to 48.3 letters in CRUISE. Furthermore, only 2% of patients in SCORE had ischemic CRVO by the previous CVOS definition of >10 DA of capillary nonperfusion [1]. An average of 42% of patients had poor baseline VA ranging from 20/125 to 20/400 and this is in contrast to CRUISE, where the numbers of patients with VA ranging from 20/200 to 20/500 in the sham, 0.3 mg, and 0.5 mg ranibizumab groups were 27%, 30.3%, and 30%, respectively. Patients with RAPD were excluded from CRUISE, but it was not an exclusion criteria in SCORE. In a recent post hoc analysis for SCORE, higher baseline visual acuities, and less severe anatomical abnormalities of the retina (center point thickness and areas of retinal hemorrhage, thickening, and fluorescein leakage) were predictive of better visual acuity outcomes. In addition with regard to center point thickness outcomes, shorter duration of macular edema was associated with better outcomes. This meant that differences in baseline VA and duration of edema between both studies might have favored better outcomes for CRUISE [19].

#### 2.1.2. Outcomes (Table 2)

The primary outcome and major end point results for the SCORE-CRVO are summarized in Table 2. The study showed that 6.8%, 26.5%, and 25.6% gained 15 letters or more at 12 months for the observation, 1 mg, and 4 mg groups, respectively. There was a change in mean visual acuity letter score of loss of 1-2 letters compared with a mean loss of 12 in the observation group. When subgroup analysis was done for patients with pseudophakia at baseline, a mean gain of 2 letters was seen in the 1 mg group compared to a loss of 1 letter in the 4 mg group and 14 letters loss in the observation group.

This is in contrast to the results of CRUISE that showed that at 6 months 46.2% and 47.7% of patients showed 15 letters or more improvement in the 0.3 mg and 0.5 mg ranibizumab groups, compared to 16.9% in the sham group. In addition, the mean letter gains were 12.7, 14.9, and 0.8 in the 0.3 mg, 0.5 mg, and the sham groups, respectively. However, it is to be noted that at 12 months the group in SCORE with baseline VA (20/80–20/100) showed the best gains with 7.8 letters gained in the 1 mg group and 2.8 letters in the 4 mg groups compared to 13 letters loss in observation. It is also to be noted that the percentage of patients with 15 letter gains was 47%, 38%, and 6% in the 1 mg, 4 mg, and observation groups, respectively. When put in context, perhaps had the differences in the baseline criteria of both studies been similar, the differences in visual outcomes could have been smaller (Table 2).

The SCORE study showed that although at month 4 the median decrease in central macular thickness was greater in the 4 mg group (196 *μ*m decrease) than the 1 mg (77 *μ*m decrease) and the observation group (125 *μ*m decrease), the percentage of patients with CMT < 250 *μ*m was similar for the 3 study groups at the end of the first year of followup. This meant that the natural history of RVO (also previously documented in the CVOS study [18]) shows a gradual decrease in macular thickness despite differences in visual outcomes [1]. However, perhaps the chronic nature of the edema leads to structural damage to the inner retina and even after edema resolved high VA cannot be achieved. This was also demonstrated in the CRUISE study which showed that the sham/observation group that once shifted to PRN Ranibizumab after 6 months showed improvement in VA but did not catch up to the 0.3 or the 0.5 mg dose in terms of BCVA at the end of the first year [9].

#### 2.1.3. Safety

The major complication during this trial was glaucoma; 35% of patients in the 4 mg triamcinolone group initiated IOP lowering medications compared with 20% in the 1 mg group and 8% in the observation group. Four patients required tube surgery in the triamcinolone group during the 24 months of the study but it was deemed by the investigator that the cause of elevated IOP was neovascular glaucoma.

Cataract was the second most common complication, 18% in the observation group compared with 26% and 33% in the 1 mg and 4 mg triamcinolone groups. No eyes in the observation or 1 mg group had cataract surgery during the first year and only 4 eyes in the 4 mg group.

### 2.2. GENEVA Study

The GENEVA study was in fact two randomized, prospective, sham controlled clinical trials that looked at the effect of using two different doses of the dexamethasone implant (0.35 mg and 0.7 mg) compared with a sham procedure group in patients with branch and central retinal vein occlusion [20]. Dexamethasone is a potent, water soluble steroid that is delivered to the vitreous cavity using an intravitreal implant (OZURDEX, Allergan, Inc., Irvine, CA). The drug-copolymer complex gradually releases the drug over several months after insertion. Although it was initially estimated that the duration of action of the drug would be 6 months, a recent large scale RCT in diabetic macular edema patients (MEAD study) showed that the actual efficacy is closer to 4 months [21]. The primary end point for GENEVA study was 6 months and patients received only a single injection during this period leaving a chance for undertreatment. The extension open label phase of GENEVA followed up patients to 12 months and allowed patients to receive a 0.7 mg DEX implant at 6 months if they met criteria for reinjection [17]. 99% of patients in all groups were reinjected meaning that a single DEX implant is ineffective at treating CRVO.

The primary outcomes were different in both GENEVA trials. One measured the proportion of eyes achieving at least a 15-letter improvement from baseline BCVA at day 180 and the other looked at the time needed to reach a 15-letter improvement from baseline. These outcomes are different from other trials such as SCORE and CRUISE, which looked only at proportion achieving 15 letters or more improvement, making it difficult for cross study comparisons [9, 16].

#### 2.2.1. Baseline Characteristics (Table 3)

Again we will cautiously try to make comparisons with CRUISE which was the major anti-VEGF trial. Attempts to compare patients based on baseline criteria and demographics are difficult because GENEVA used a combined pool of both BRVO and CRVO patients. In addition, there were twice as many patients with BRVO (66%) than CRVO (34%). Nonetheless, the mean baseline VA was approximately 54 letters in the 3 groups compared with 48 letters in CRUISE. The average duration of edema was 5.2 months in GENEVA compared with 3.3 months in CRUISE. This is important because a recent post hoc analysis of data from GENEVA showed that each one-month increase in ME duration was associated with a significantly lower likelihood of achieving better visual outcomes at 6 and 12 months [22]. This association was stronger in BRVO patients but was found to be weaker in CRVO patients and was not statistically significant. This is in line with the data from SCORE that found that chronicity in CRVO was not a predictive factor for VA outcomes [19]. In addition, the percentage of patients having edema less than 3 months was 15.3%–18%, compared to 69% in CRUISE indicating that patients in the GENEVA study had a more chronic edema [9, 20].

#### 2.2.2. Outcomes

At 6 months, the cumulative response rate (time to achieve 15 letters of improvement from baseline) in patients with both BRVO and CRVO was 41% in the 0.7 mg group, 50% in the 0.35 mg group, and 23% in the sham group. At day 180, the proportion of eyes achieving 15-letter improvements in the DEX group (22%) was not statistically different than the sham group (18%). However there was a significant difference between them from day 30 to day 90 that disappeared as they reached the 180-day followup. The mean increase from baseline VA was significantly greater in both DEX implant groups than sham with the greatest difference between them at day 60 (7 letters).

Subgroup analysis for CRVO patients showed there was a significant difference between mean change in BCVA in the DEX implant groups, compared to the sham group on days 30, 60, and 90 of followup. However, at 6 months there was no significant difference between the different groups with mean gain of 2 letters in the 0.35 mg group, 0 letters in the 0.7 mg group, and −2 letters in the sham group. The peak letter gain was approximately 10 letters in the 0.35 mg group and 9 letters in the 0.7 mg group at day 60. In addition, the percentage of patients showing 15-letter improvements was significantly higher in the 0.35 mg and 0.7 mg doses compared to sham on days 30, 60, and 90 reaching a peak at day 60 with 33% of patients in the 0.35 mg group, 29% in the 0.7 mg group, and 9% in the sham group. However, by day 180, the differences were not significant (18% in the 0.7 mg group, 17% in the 0.35 mg group, and 12% in sham group).

It would appear that the maximum VA gains are achieved at 60 days and that these gains are gradually lost as the drug loses efficacy over time. These fluctuations were also seen during the extension phase after the second injection that showed a peak in BCVA around the 240-day mark that gradually decreased to preinjection levels at 360 days [17]. The DEX implant seems to have a short duration of action and by 6 months was no longer effective. Had patients been allowed a second injection at 4 months or injections were guided based on an OCT fluid based strategy final visual outcomes might have been higher.

A post hoc analysis showed that the duration of ME at baseline affected final visual outcomes; eyes with a shorter duration of ME (<90 days) showed greater response than patients with longer duration of ME. At day 60, the number of patients achieving more than 15-letter improvement was 38% in the 0.7 mg group and 35% in the 0.35 mg group in eyes with edema < 90 days, compared to 27% in both DEX groups in eyes with edema > 90 days. However in the subgroup analysis, patients with CRVO did not show any difference in treatment response with regard to the duration of edema. These changes were also mirrored in the post hoc analysis of SCORE [19]. Perhaps these differences indicate that with regard to steroids the duration of CRVO is not a predictive factor to final visual outcomes. Another possible explanation is that steroids might have a potent effect on chronic edema as demonstrated by the FAME study for DME [23]. Therefore, with equal potency for acute and chronic edema, outcomes would be similar regardless of duration. However, these results seem to be exclusive for CRVO and not BRVO. There have been no large RCTs comparing the efficacy of anti-VEGF in patients with short and long durations of edema in patients with CRVO. This means we still have unanswered questions as to whether steroids are the better treatment option in patients with chronic CRVO.

#### 2.2.3. Safety

The only adverse events that occurred significantly more frequently in the DEX implant treatment groups than the sham group were ocular pain, ocular hypertension, and anterior chamber cells.

There was no significant difference between the incidence of cataract in the 0.7 mg DEX implant group (7.3%), 0.35 mg DEX implant group (4.1%), and sham group (4.5%). Only 2 patients had cataract extraction during the study. In the 12-month extension, patients who received more than 2 injections had no differences in adverse events compared to the group that received only one implant except for cataract [17]. The incidence of cataract adverse events (AEs) was 29.8% (90/302 DEX 0.7/0.7 mg group) and 19.8% (56/283 DEX 0.35/0.7 mg group) in patients receiving 2 DEX implants. This was higher compared to the group that received only a single injection during the first year, with 7.6% (5/66) and 7.7% (6/78) developing cataract in phakic patients receiving the 0.7 mg DEX implant and 0.35 mg DEX implant groups, respectively. In the group that did not receive any injections at all the rate of cataract adverse events was 5.7% (5/88) but the patient numbers were much lower.

In the MEAD study for DME, patients were divided into 3 groups: 0.35 mg DEX implant, 0.7 mg DEX implant, and sham [21]. Patients were then followed up for 36 months and that gave an opportunity for long term complications to be examined noting of course that the mean number of treatments in the MEAD study for the 0.7 mg and 0.35 mg group was 4-5 versus 2 injections only in the GENEVA extension study. In the MEAD study, unlike GENEVA, there was a higher percentage of cataract formation with 67.9%, 54.1%, and 20.4% in the 0.7 mg implant, 0.35 mg implant, and sham groups, respectively. In addition cataract surgery rates were 59.2%, 52.3%, and 7.2% in the 0.7 mg, 0.35 mg, and the sham groups, respectively. This higher percentage can be explained by the longer duration of followup and the repeated treatments.

With regard to ocular hypertension, there was a significant difference between the DEX implant groups and the sham group. The intraocular pressure peaked at day 60 which also correlated with the peak visual gains achieved by patients in the DEX group. However, at day 180 there was no significant difference between the different groups. This trend continued during the extension study with patients receiving the second injection again peaking at day 240 before dropping at day 360 [17]. At the end of the study, the percentage of eyes receiving IOP lowering medications in the DEX implant group was 24% at 6 months, with an additional 10.3% at the end of 12 months [17, 20]. Six eyes in the group that received 2 DEX implants and 6 eyes in the single DEX group required laser or IOP reducing surgeries of which 4 had neovascular glaucoma. It is interesting that the peak IOP correlated with the peak improvements in VA, which probably meant the IOP increase was drug related and with the diminishing effect of the implant IOP improved. The MEAD study [21] for DME showed similar results with one-third of patients in each DEX implant group showing an increase in IOP requiring treatment during the study. However, as in GENEVA very few patients required incisional surgery for IOP reduction (0.3%). Mean IOP also showed similar fluctuations as in GENEVA where IOP peaked close to 3 months before returning to baseline levels at 6 months. Because of the long term followup, it was found that in MEAD [21] the incidence of IOP related adverse events remained the same from year 1 to year 3 of the study with no apparent increase. Interestingly the same number of patients using IOP lowering medications remained the same from year to year. This could lead us to several conclusions. First, the effect of DEX implant on IOP did not seem cumulative. Secondly, it would seem that only a certain percentage of patients seem to be affected and those who are not susceptible do not witness IOP increases later with chronic use. In addition, IOP increase is dependent on the effects of dexamethasone and once its levels decrease in the vitreous, intraocular pressure normalizes. Lastly the vast majority of patients can be controlled by IOP lowering medications.

## 3. Steroids versus Anti-VEGF

No large randomized studies have compared between steroids and anti-VEGFs. A small study by Gado AS and Macky TA was conducted on 60 patients with nonischemic CRVO in which patients were randomized into two groups, one receiving bevacizumab and the other receiving Ozurdex implants [24]. The study showed that there was no significant difference in BCVA or macular thickness between the two groups at 6 months. However, there was a significantly higher IOP in the dexamethasone group. Another study by Ding et al., conducted on 32 patients, showed similar results at 9 months [25]. A third study by Chiquet et al., conducted on 102 patients randomized to receive anti-VEGFs and dexamethasone implants showed that at 3 months there was significantly better visual outcomes in the DEX group with no difference in the CMT. However, these differences were not maintained and by the first year there was no difference in anatomical or visual outcomes. In addition elevated IOP > 21 mmhg was more frequent in the DEX group (21%) compared to the anti-VEGF group (3%, *P* = 0.008) [26].

It is possible that the different outcomes of the three major RCTs (CRUISE, SCORE, and GENEVA) can be partly explained by differences in the initial baseline criteria. A recent study by Thom et al. attempted to compare between trials using a combination of multinomial and indirect Bayesian comparison models [27]. It showed that there was a trend for greater ranibizumab associated visual gains compared with dexamethasone at months 1 and 6 in a common clinical context, although results were not classically significant.

## 4. Steroids as Adjunct or Alternative Therapy to Anti-VEGF

The difference in visual outcomes (or lack thereof in certain cases), as well as the higher incidence of complications, means that anti-VEGF would be the more preferable first line drug. Nonetheless, steroids can be used as a second-line drug in resistant cases or as an adjunct from the start. A retrospective study by Sharareh et al. looked at 18 patients categorized as complete or partial responders to bevacizumab that were given dexamethasone implants [28]. The study showed that both subgroups responded with an improvement in both central macular thickness (average 147 micrometers) and visual acuity (mean improvement of 0.25 logMAR). The OMAR study compared between the effects of Ozurdex and triamcinolone acetonide in cases of refractory cystoid macular edema despite repeated bevacizumab therapy due to retinal vein occlusion [29]. It showed that adding steroids improved central macular thickness significantly (*P* < 0.0001). However, final BCVA did not change significantly after steroid introduction (*P* = 0.06). There was no difference between TA and DEX regarding anatomic or functional outcomes.

As an adjunct, a case series by Singer et al. showed that dexamethasone implant with bevacizumab showed a synergistic effect in CRVO and BRVO patients, increasing VA and prolonging the time between injections, compared to either of these medications alone [30]. At 6 months, 64% of patients had a maximum visual acuity gain of 3 lines compared to baseline and had a mean increase of 16.8 letters. Also, 18% of patients did not require reinjection. Another study by Maturi et al. compared between patients who received bevacizumab alone and patients who received combination therapy with dexamethasone implants [31]. At 6 months, there was a greater reduction in mean CMT in the combined group compared to the monotherapy group, despite no significant differences in VA. However several studies comparing between the effects of anti-VEGFs and combined anti-VEGFs and triamcinolone found no significant difference in BCVA or CRT between the two subgroups [25, 32–34].

These data show that dexamethasone implants and, to a lesser extent, TA may be a suitable option in resistant cases as monotherapy or as adjunct therapy to ranibizumab or bevacizumab. However more large scale RCTs are needed to show true benefit of this treatment modality.

## 5. Summary

As monotherapy, steroids alone have not been proven to be superior to anti-VEGFs. With the higher incidence of side effects, especially after repeated dosing, anti-VEGFs still remain the first-line drug for treating CRVO. As an initial therapy, combined steroids and anti-VEGFs in CRVO have not proven any advantage over anti-VEGFs alone and as such cannot be recommended based on current data. Nonetheless, the role of steroids as an alternative therapy in resistant cases (alone or in combination with anti-VEGFs) remains a viable option. Dexamethasone and triamcinolone both appear as viable options in resistant cases.

In conclusion, there remains a limited role for steroids in current practice. More RCTs are needed to truly evaluate its efficacy in resistant cases.

## Figures and Tables

**Table 1:** Baseline criteria comparison between the CRUISE and SCORE studies.

	CRUISE [9]	SCORE [16]
Duration of edema (mean)	3.3 months	4 months
Percentage having edema <3 months	69%	29%
Mean baseline VA	48.3 letters	51 letters
Patients with poor VA	29% (20/200–20/500)	42% (20/125–20/400)
Ischemic CRVO	0.5%	2%

VA: visual acuity; CRVO: central retinal vein occlusion.

**Table 2:** Comparison between CRUISE and SCORE with regards to visual outcomes.

	CRUISE [9]	SCORE [16]		
		Overall	Pseudophakics	Baseline VA (20/80–20/100)
15 letter gains	45.5% (0.3 mg)	26.5% (1 mg)	NS	47% (1 mg)
	46.2% (0.5 mg)	25.6% (4 mg)		38% (4 mg)

Mean change in VA	13.9 gain letters	1-2 letter loss	2 letter gain (1 mg)	7.8 letters gain (1 mg)
	(0.3 mg and 0.5 mg)	(1 mg and 4 mg)	1 letter loss (4 mg)	2.8 letters gain (4 mg)

VA: visual acuity.

**Table 3:** Baseline criteria differences between CRUISE and GENEVA.

	CRUISE	GENEVA
Mean VA	48 letters	54 letters
Duration of edema (months)	3.3 months	5.2 months
Patients with edema <3 months	69%	15.3%–18%
